# Rumen-Protected Fat and Rumen-Protected Choline Co-Supplementation: Impacts on Performance and Meat Quality of Growing Lambs

**DOI:** 10.3390/vetsci12060525

**Published:** 2025-05-28

**Authors:** Haitao Liu, Fadi Li, Fei Li, Zhiyuan Ma, Tao Wang, Qinwu Li, Xinji Wang, Kaidong Li

**Affiliations:** 1 State Key Laboratory of Herbage Improvement and Grassland Agro-Ecosystems, College of Pastoral Agriculture Science and Technology, Lanzhou University, Lanzhou 730070, China; liuht19@lzu.edu.cn (H.L.); lfei@lzu.edu.cn (F.L.); mzy@lzu.edu.cn (Z.M.); taowang2023@lzu.edu.cn (T.W.); liqw2023@lzu.edu.cn (Q.L.); 2 Animal Husbandry and Veterinary Station, Wuwei 733399, China; cmlht@163.com; 3 Agricultural and Rural Bureau Zhongxing Town Animal Husbandry and Veterinary Station, Wuwei 733399, China; 18419166373@163.com

**Keywords:** fatty acid, lambs, meat quality, rumen-protected choline, rumen-protected fat

## Abstract

This study aimed to assess the effect of co-supplementing rumen-protected fat and rumen-protected choline on growth performance, carcass traits, and meat quality in lambs. Using a randomized experimental design, 45 weaned female Tian×Hu crossbred lambs (3 months old; average body weight: 27.34 ± 0.57 kg, mean ± SD) were randomly allocated to one of three dietary treatment groups. The three dietary treatments were as follows: a basal diet group (CON), a group receiving 2% rumen-protected fat in place of 2% barley (RPF), and a group supplemented with 2% rumen-protected fat and 0.4% rumen-protected choline, replacing 2% barley and 0.4% corn germ in the basal diet (RPFC). Compared to the CON group, neither the RPF nor RPFC treatments resulted in significant differences in growth performance (*p* > 0.05). However, the RPFC group showed a 5.3% increase in dry matter intake (DMI) compared to the RPF group (*p* < 0.05). Compared with the CON, the RPF treatment increased 69.23% the relative abundance of C18:2n-6t (*p* < 0.05), but the content of C17:0, C17:1, C18:1n-9c, and iso-C18:0 in *Longissimus lumborum* was decreased by 16.49%, 15.78%, 6.45% and 27.78%, respectively (*p* < 0.05). The RPFC treatment increased the relative abundance of C16:1 in *Longissimus lumborum* (*p* < 0.05). The RPF and RPFC treatments significantly increased serum levels of high-density lipoprotein (HDL) and total cholesterol compared to the CON group (*p* < 0.05). The RPF treatment raised HDL by 50.00% and total cholesterol by 38.03%, while the RPFC treatment increased HDL by 39.47% and total cholesterol by 26.03%. Furthermore, compared to the RPF group, the RPFC treatment led to a 13.47% increase in the 45 min b* color value of the *Longissimus lumborum* (*p* < 0.01) and a significant 45.45% reduction in the relative abundance of C18:2n-6t fatty acid in the same muscle (*p* < 0.05). In summary, rumen-protected choline reduces the negative effects of rumen-protected fat on feed intake in lambs and changes fatty acid profile in meat.

## Simple Summary

Choline enhances energy utilization and redirects fatty acids toward beneficial pathways. This study investigated the effects of supplementing growing lambs with rumen-protected fat and rumen-protected choline on performance and meat quality. Co-supplementation increased feed intake and elevated levels of the C16:1 fatty acid in lamb meat. These changes were linked to the role of choline in regulating lipid metabolism, which highlights that RPF and RPC co-supplementation improve both productivity and nutritional value in lamb production.

## 1. Introduction

An optimal alternative feeding system for lambs is crucial for maximizing production scale and economic efficiency in sheep farming [1]. Directly adding fat to the diet may result in its association with dietary fiber in the rumen, which can interfere with microbial access to the fiber and hinder fermentation [1]. This interference disrupts fiber digestion and impairs ruminal fermentation [2]. This issue can be effectively addressed using rumen protection techniques, which help maintain normal rumen function while increasing the dietary energy density [3,4]. The main types of rumen-protected fat include hydrogenated fats [5], fatty acid amides [6,7], calcium salts of fatty acids [8], and other types. Achieving optimal results requires careful control of fat concentration, with typical inclusion levels for lambs ranging from 2% to 5% [9,10,11].

Metabolic choline derivatives are structural components of membranes, neurotransmitters, and mediators of hepatic lipid metabolism [12]. It positively affects beef cattle and lambs’ growth performance and carcass characteristics [13]. Therefore, it is crucial to supplement ruminant diets with adequate choline to support optimal fat metabolism [14]. Low levels of rumen-protected choline supplementation (0%, 0.1%, 0.2%, and 0.3%) have shown minimal effects on growth performance and carcass traits in lambs [15]. However, other studies have reported that supplementing with 0.25% DM of rumen-protected choline can improve growth performance by modulating blood lipid profiles and fatty acid metabolism in skeletal muscle, ultimately improving meat quality [16,17].

On the other hand, higher inclusion levels (e.g., 0.75% DM) have been associated with reduced body weight gain [16]. Rumen-protected choline has also been found to promote hepatic triacylglycerol secretion, function as a lipotropic agent, and support hepatic fat metabolism in ruminants [18,19,20]. This nutritional approach may aid in the more efficient mobilization of adipose tissue to meet energy demands during key growth periods while favoring muscle development over fat deposition. Therefore, further in vivo studies are warranted to explore the combined effects of rumen-protected fat and choline supplementation on blood biochemical parameters and meat quality in lambs.

This study hypothesized that the combined supplementation of rumen-protected fat and rumen-protected choline would synergistically improve lipid metabolism in lambs. Accordingly, the objective was to evaluate the effects of this co-supplementation on growth performance, carcass characteristics, and meat quality.

## 2. Materials and Methods

The experimental protocols and procedures for this study were approved by the Animal Care and Use Committee of the College of Pastoral Agriculture Science and Technology, Lanzhou University (Approval No. CPAST-Li-2024-035).

### 2.1. Experimental Design and Treatments

Using a randomized experimental design, 45 female Tian×Hu crossbred lambs, 3 months old with a similar body weight (27.34 ± 0.57 kg, mean ± SD), were randomly assigned to one of three dietary treatments. The basal diet met 90% of the nutritional requirements for lambs weighing 30 kg and demonstrated a growth rate of 300 g/d, according to NRC guidelines (2007). The three treatments were the control treatment (CON) with basal diet, the RPF treatment with 2% rumen-protected fat replacing 2% barley in the basal diet, and the RPFC treatment with 2% rumen-protected fat and 0.4% rumen-protected choline replacing 2% barley and 0.4% corn germ in the basal diet (Table 1).

### 2.2. Animal Feeding Management

Before the start of the experiment, the pens were thoroughly cleaned and sanitized. During the 7-day adaptation period (when the basal diet was provided) and the subsequent growth experiment (with the designated diets), all lambs were fed twice daily at 8:00 and 17:30, with ad libitum access to feed and water. On days 1 and 64 of the growth experiment, all lambs underwent a 12 h fast before being weighed. Feed consumption for each lamb was recorded throughout the experiment to calculate average daily gain (ADG) and feed conversion (F/C). ADG was calculated as (Final Body Weight − Initial Body Weight)/Duration of Feeding, while F/C was calculated as Total Feed Intake/(Final Body Weight − Initial Body Weight).

### 2.3. Hematological Sampling and Determination

One day following the growth experiment, all lambs were fasted for 12 h, after which 5 mL blood samples were collected via the jugular vein. The blood samples were centrifuged at 3000 rpm for 5 min, and the plasma was harvested and stored at −20 °C for subsequent biochemical analysis. Hematological parameters were measured using commercial ELISA kits, which included total cholesterol (TC) (Code: E-EL-0064), triglycerides (TG) (Code: MBS2601343), high-density lipoprotein cholesterol (HDL-C) (Code: CSB-E15854m), low-density lipoprotein cholesterol (LDL-C) (Code: E-EL-0066), glucose (GLU) (Code: 10009582), urea (UREA) (Code: DIUE-100), creatinine (CREA) (Code: E-EL-0062), free fatty acids (FFA) (Code: 999-34691), and beta-hydroxybutyrate (β-HB) (Code: 700190). All ELISA kits were sourced from Mindray Bio-Medical Electronics Co. Ltd. (Shenzhen, China).

### 2.4. Carcass Characteristics in Lambs

Three days after the 63-day collection period, 10 lambs were randomly selected from each group. Following a 12 h fast, the lambs were stunned using an electric current (200 V for 4 s) and then slaughtered. According to Winders et al. (2022) [15], the following carcass traits were systematically measured and recorded: carcass weight, dressing percentage, subcutaneous backfat thickness, rib grading GR value (defined as the fat thickness measured at the 12th thoracic rib perpendicular to the outer carcass surface, expressed in millimeters), caudal adipose tissue mass (tail fat), omental adipose tissue mass (peritoneal fat), visceral adipose tissue mass, and the total dissectible adipose tissue mass (the sum of all measured fat depots). Further carcass traits measured and recorded included live weight, backfat thickness, GR, perirenal fat, abdominal fat, tail fat weight, and the combined weight of these fat depots.

### 2.5. Assessment of Meat Quality Parameters

Two sub-samples of the *Longissimus lumborum* were collected from each lamb. One sample was used to determine pH, drip loss, and meat color characteristics. Another sample was vacuum-packed and stored at −80 °C for subsequent fatty acid (FA) and ether extract (EE) analysis. The pH was measured after 45 min and 24 h using a pH meter for meat (PHSJ-5, Shanghai Leici Instrument Co., Ltd., Shanghai, China). Drip loss, *Longissimus lumborum* area, and EE content were evaluated following the protocols recommended by Gurgeira et al. [16]. Meat color was determined using a Minolta CR-400 chroma meter (Konica Minolta, Tokyo, Japan).

Muscle samples for fatty acid (FA) measurement were stored at −80 °C and freeze-dried within one month of collection. A 0.2 g portion of the freeze-dried muscle sample was transferred into a 16 × 125 mm screw-capped tube, adding 0.7 mL of KOH aqueous solution and 6.3 mL of methanol (Bioteke, Beijing, China). The tube was then placed in a 55 °C water bath for 1.5 h, with manual agitation every 20 min for 5 s to promote sample permeation, dissolution, and hydrolysis. After the mixture cooled to room temperature, 0.58 mL of H_2_SO_4_ aqueous solution (Bioteke, Beijing, China) was added. The tube was mixed by inversion, allowing K_2_SO_4_ to precipitate, and then returned to the 55 °C water bath for an extra 1.5 h with the same agitation schedule. The tube was placed in an ice bath after forming fatty acid methyl esters (FAMEs). An internal standard (2 mL of a solution containing 1 g of C21:0 in 1 L hexane) was added, and the tube was vortexed for 5 min before centrifugation. The hexane layer containing the FAMEs was then transferred to a GC vial for analysis using gas chromatography (1600, Thermo Fisher Scientific, Waltham, MA, USA) with a flame ionization detector and a DB-23 column (60 m × 0.25 mm × 0.25 µm). The injection port temperature was set to 220 °C with a 9:1 split ratio. The initial column temperature was held at 120 °C for 5 min, then increased at a rate of 3 °C per minute to 200 °C, where it was held for 10 min, followed by a final ramp to 240 °C at 1.5 °C per minute. The total run time was 78.333 min, with helium as the carrier gas. The concentration of long-chain fatty acids is calculated using the following formula:

CFA = (A_FA_ × C_IS_ × RF_FA_)/(A_IS_ × Wsample)

where

- C_FA_ is the target fatty acid concentration in the sample (mg/g muscle tissue).
- A_FA_ is the chromatographic peak area of the target fatty acid.
- A_IS_ is the peak area of the internal standard (C21:0).
- C_IS_ is the concentration of the added internal standard (μg).
- RF_FA_ is the relative response factor of the target fatty acid (determined by standard calibration).
- W sample is the weight of the muscle sample (g).

The calibration formula for determining the relative response factor is as follows:

RF_FA_ = (A_FA_ × C_std_)/(A_std_ × C_FA_)

where

- RF_FA_ is the relative response factor of the target fatty acid.
- A_FA_ is the peak area of the target fatty acid in the standard.
- C_std_ is the concentration of the internal standard in the calibration solution (μg/mL).
- A_std_ is the peak area of the internal standard.
- C_FA_ is the concentration of the target fatty acid standard (μg/mL).

### 2.6. Statistical Analysis

Data were analyzed using a general linear model in SPSS23, with treatment as a fixed factor. Before conducting ANOVA, normality was checked using the Shapiro–Wilk test, and homogeneity of variances was assessed with Levene’s test. The treatment effect was evaluated through one-way ANOVA, followed by Duncan’s post hoc multiple comparisons at α = 0.05.

## 3. Results

### 3.1. Growth and Carcass Characteristics

No difference in DMI was observed between the PRF and RPFC treatments compared to the CON treatment (*p* > 0.05, Table 2). However, the RPFC treatment resulted in a 5.3% increase in DMI compared to the RPF treatment (*p* < 0.01). No significant differences in carcass characteristics were found among the three treatments (*p* > 0.05, Table 3).

### 3.2. Effects on the Serum Biochemical Indices

Compared to the CON treatment, the RPF treatment increased HDL-C and TC by 50% and 38.03%, respectively, while the RPFC treatment led to increases of 39.47% in HDL-C and 26.03% in TC (*p* < 0.01, Table 4). No significant differences were observed in other indices (*p* > 0.05).

### 3.3. Effects on Meat Quality Parameters

Forty-five minutes post-slaughter, the b* value was 13.47% higher in the RPFC treatment compared to the RPF treatment (*p* < 0.01, Table 5), while no significant difference was observed when compared to the CON treatment (*p* > 0.05). Twenty-four hours after slaughter, no significant differences in meat color were found among the three treatments (*p* > 0.05). Moreover, the *Longissimus lumborum* area was significantly reduced (*p* < 0.01) in the RPF (−22.92%) and RPFC (−17.80%) treatments compared to the CON treatment. No significant difference in drip loss was observed among treatments (*p* > 0.05).

Compared to the CON treatment, the RPF treatment increased the relative abundance of C18:2n-6t (+69.23%; Table 6) but decreased the levels of C17:0 (−16.49%), C17:1 (−15.78%), C18:1n-9c (−6.45%), and iso-C18:0 (−27.78%) in the *Longissimus lumborum* (*p* < 0.05). Moreover, the RPFC treatment significantly increased (*p* < 0.05) the content of C16:1 (+12.33%) and ∑SFA (+5.04%) while reducing the ∑MUFA/∑SFA ratio (−9.72%) in muscle compared to the CON treatment. The C18:2n-6t content in muscle was 45.45% lower in the RPFC treatment than in the RPF treatment (*p* < 0.05).

## 4. Discussion

Generally, dry matter intake tends to decrease when additional fat is included in an animal’s diet [21], which can impact the rumen digestion of fiber [22]. While fat has a higher energy density than carbohydrates [23], it is not an essential nutrient for rumen microbes [24]. Nigdi et al. also noted a reduction in DMI when fat was added to the basal diet [25]. In this study, there was no significant change in DMI in the RPF group compared to the CON group, likely due to the lower inclusion level of RPF. Previous studies have shown that various types of RPFs (such as prilled fat, prilled fat with lecithin, and calcium soap) had no significant effects on final body weight, total weight gain, average daily gain (ADG), or DMI in sheep [26]. Similar findings were reported by Behan et al. [26].

Furthermore, adding 0.25% RPC to the basal diet of lambs did not significantly affect feed intake [16], which aligns with the current study’s results. DMI was significantly higher in the RPFC group than in the RPF group. This increase in DMI may be attributed to the role of choline in lipid metabolism and hepatic fat transport, as it serves as a precursor for the synthesis of phosphatidylcholine and trimethylglycine [27,28]. DMI was a trend toward lower in the PRF group and a trend toward higher DMI in the PRFC group compared to the control group. This suggests that choline can mitigate the adverse effects of protected rumen fat on the DMI of lambs.

Generally, the inclusion of RPF has an evident effect on blood indices. Previous studies have reported that adding RPF to the basal diet of bulls significantly increased blood levels of TC and HDL-C [29,30], which aligns with the findings of this study. This effect may be attributed to the fatty acids in rumen-protected fats, which enhance the activity of cholesteryl ester transfer protein, therefore promoting cholesterol transport and elevating HDL cholesterol levels [31]. It is important to note that as the concentration of RPF increases, blood cholesterol levels also rise [32], further supporting the idea that dietary fat supplementation can promote lipid metabolism in the blood [24]. Rodríguez-Guerrero et al. found that choline supplementation significantly raised blood glucose and cholesterol levels in lambs [33], which is consistent with the current study. However, unlike their findings, the difference in glucose levels between the RPFC and CON groups in this study was insignificant, although it tended to increase. This discrepancy may be attributed to the different forms of choline used. Both the RPF and RPFC groups significantly increased the levels of HDL-C and TC compared with the CON group. Notably, there was a trend toward lower levels of HDL-C and TC in the RPFC group compared to the RPF group. In addition, a 0.50% supplementation of RPC has been shown to reduce HDL-C concentration [16]. This suggests that rumen-protected choline can reduce the blood lipid.

Meat color is a critical factor affecting consumer purchasing decisions and is commonly used to indicate meat quality [34]. Several studies have reported that RPF supplementation does not significantly affect b* values [35,36,37], which is consistent with the findings of this study. Furthermore, choline is a key precursor for synthesizing phosphatidylcholine (lecithin), an essential component of lipid metabolism [38]. Choline supplementation has been shown to enhance lipid metabolism in muscle tissue and increase b* values [39]. In the present study, the b* value at 45 min post-slaughter was a trend toward lower in the PRF group and a trend toward higher in the PRFC group compared to the control group, and the b* value at 45 min post-slaughter was significantly higher in the RPFC group than in the RPF group, likely due to the influence of choline.

The *Longissimus lumborum* area is an essential metric for evaluating carcass yield in lambs. Previous research indicated that supplementing lamb diets with 0.25%, 0.50%, and 0.75% RPC improved meat quality at 0.25% but exerted negative effects at 0.75% [16]. In this study, the RPFC treatment significantly reduced the *Longissimus lumborum* area compared to the CON group, potentially due to the higher level of RPC used. Furthermore, RPC supplementation at doses of 5 g/kg or lower has shown limited benefits [40]. Highlighting the importance of carefully optimizing RPC dosages for practical applications.

In recent years, fatty acids have gained significant attention as key indicators of meat quality and nutritional value. As a common saturated fatty acid, elevated C16:0 levels may be attributed to enhanced hepatic fatty acid synthesis or increased absorption of dietary fats [14]. The reduction in C17:0 and C17:1 may reflect alterations in intestinal microbial metabolism, as these fatty acids are typically associated with intestinal microbial processes [27]. Furthermore, the present study found that RPF supplementation reduced the levels of C18:1n-9c and iso-C18:0, both of which are critical intermediates in the biohydrogenation process [41]. This suggests that RPF supplementation may inhibit the biohydrogenation of UFA in ruminants, thus affecting meat quality and nutritional composition. Previous research has also shown that adding RPF to ruminant diets can decrease the hydrogenation of UFA in the rumen, leading to reduced formation of SFA, such as stearic acid, and enhanced deposition of unhydrogenated UFA within muscle tissue [42].

Moreover, adding 2% RPF to the diet significantly increased the concentration of C18:2n-6t in muscle tissue, consistent with the findings reported by Razzaghi et al. [43]. However, research investigating the effects of RPF and RPC on lamb meat’s fatty acid profile, particularly their synergistic interaction, remains limited. In the present study, the concentration of C18:2n-6t was lower in the RPFC group compared to the RPF group alone. This reduction may be attributed to the ability of RPC to promote lipolysis and fatty acid oxidation, reducing fat deposition and the synthesis of trans-fatty acids [44]. This difference may also reflect the synergistic effects of RPF and RPC on trans-vaccenic acid levels, although the exact mechanisms underlying their interaction require further investigation.

## 5. Conclusions

In conclusion, rumen-protected choline reduces the negative effects of rumen-protected fat on feed intake in lambs and changes fatty acid profile in meat.

## Figures and Tables

**Table 1:** Ingredients and proximate composition of experimental diets.

Ingredients, g/kg	CON	RPF	RPFC
Corn straw	150	150	150
Barley	600	580	580
Molasses	30	30	30
Corn germ feed	45	45	41
Soybean meal	67	67	67
Cottonseed meal	50	50	50
NaHCO_3_	10	10	10
Rumen-protected fat ^1^	0	20	20
CaCO_3_	13	13	13
Ca(HCO_3_)_2_	5	5	5
NaCl	5	5	5
Gelatinized starch-urea ^2^	6	6	6
Rumen-protected choline ^3^	0	0	4
CaSO_4_	2	2	2
Yeast culture	10	10	10
Premix ^4^	5	5	5
Total	1000	1000	1000
Proximate composition, kg/100 kg			
DM	89.89	90.63	91.32
CP	16.22	16.97	15.88
NDF	29.36	28.78	28.11
ADF	11.95	11.92	12.56
EE	2.71	3.21	3.61
Ash	7.97	8.77	8.65
ME, MJ/kg	10.30	10.32	10.32

^1,2,3,4^ Gansu Runmu Bioengineering Company manufactures all products. ^4^ Premix: S, 200 mg/kg; Fe, 25 mg/kg; Zn, 40 mg/kg; Cu, 8 mg/kg; I, 0.3 mg/kg; Mn, 40 mg/kg; Se, 0.2 mg/kg; Co, 0.1 mg/kg, VA, 940 IU/kg; VE, 20 IU/kg; VD, 132 IU/kg, Bentonite, 2 g/kg. ^1^ The rumen-protected fat is palm oil. Its fatty acid composition is as follows: C14:0 (2–3%), C16:0 (>85%), C18:0 (6–8%), and C18:1 (3–5%).

**Table 2:** Effects of RPF and RPFC on the growth performance of lambs.

Item	CON	RPF	RPFC	SEM ^4^	*p*-Value
Initial body weight, kg	26.95	27.54	27.53	0.57	0.89
Final body weight, kg	42.36	41.63	41.51	0.68	0.10
ADG ^1^, g/d	245	224	222	1.05	0.08
DMI ^2^, kg/d	1.36 ^ab^	1.32 ^a^	1.39 ^b^	0.01	<0.01
F/G ^3^	5.55	5.89	6.26	0.14	0.13

^1^ ADG, average daily weight gain. ^2^ DMI, dry matter intake. ^3^ F/G, feed to gain. ^4^ SEM, standard error of the mean. Within the same row of the table, data labeled with different lowercase letters denote statistically significant differences between the groups (*p* < 0.05). However, data marked with the same or no letter indicate that the differences are not statistically significant (*p* > 0.05).

**Table 3:** Effects of RPF and RPFC on carcass characteristics of lambs.

Item	CON	RPF	RPFC	SEM	*p*-Value
Carcass weight, kg	25.33	23.53	23.84	0.41	0.16
Live body weight pre-slaughter, kg	45.71	43.58	43.76	0.61	0.31
Dressing percentage, %	55.38	53.78	54.47	0.33	0.28
Backfat thickness, mm	3.01	3.10	2.69	0.08	0.10
GR (Carcass fat content), mm	3.54	3.39	3.11	0.08	0.08
Tail fat, kg	0.34	0.29	0.30	0.02	0.38
Perirenal fat, kg	1.11	0.76	0.99	0.07	0.10
Abdominal fat, kg	1.16	0.94	1.09	0.05	0.21
Tail fat+ Perirenal fat+ Abdominal fat, kg	2.61	1.99	2.38	0.11	0.07
(Tail fat + Perirenal fat+ Abdominal fat)/live body kg/100 kg	6.16	4.78	5.73	0.21	0.06
(Tail fat+ Perirenal fat+ Abdominal fat)/carcass, kg/100 kg	10.30	8.45	9.98	0.35	0.17

**Table 4:** Effects of RPF and RPFC on serum biochemical indices of lambs.

Item ^1^, μmol/L	CON	RPF	RPFC	SEM	*p*-Value
β-HB	235.30	266.40	288.46	24.25	0.37
FFA	528.24	636.03	582.01	46.73	0.12
CREA-S	71.93	67.74	74.21	3.30	0.38
Glu-G	3.39	3.27	3.55	0.14	0.55
HDL-C	0.76 ^a^	1.14 ^b^	1.06 ^b^	0.07	<0.01
LDL-C	0.67	0.83	0.75	0.07	0.16
TC	1.63 ^a^	2.25 ^b^	2.06 ^b^	0.14	<0.01
UREA	11.66	11.02	10.59	9.59	0.55
TG	0.23	0.26	0.25	0.28	0.14

^1^ β-HB: β-hydroxybutyrate; FFA: Free fatty acids; CREA-S: Creatinine; Glu-G: Glucose; HDL-C: High-density lipoprotein cholesterol; LDL-C: Low-density lipoprotein cholesterol; TC: Total cholesterol; UREA: Urea; TG: Triglycerides. Within the same row of the table, data labeled with different lowercase letters denote statistically significant differences between the groups (*p* < 0.05). However, data marked with the same or no letter indicate that the differences are not statistically significant (*p* > 0.05).

**Table 5:** Effects of RPF and RPC on quality indicators in *Longissimus lumborum* of lambs.

Item	CON	RPF	RPFC	SEM	*p*-Value
45 min pH	6.32	6.23	6.28	0.04	0.71
24 h pH	5.20	5.22	5.20	0.01	0.62
45 min L*	29.48	28.97	29.29	0.24	0.72
45 min a*	16.25	15.85	16.27	0.22	0.06
45 min b*	6.08 ^ab^	5.79 ^a^	6.57 ^b^	0.11	<0.01
24 h L*	34.81	34.95	35.50	0.32	0.65
24 h a*	20.39	20.35	20.12	0.22	0.87
24 h b*	14.14	13.65	13.50	0.18	0.34
Drip loss, %	2.39	2.74	2.24	0.20	0.59
*Longissimus lumborum* area, cm^2^	18.93 ^b^	14.59 ^a^	15.56 ^a^	0.56	<0.01

Within the same row of the table, data labeled with different lowercase letters denote statistically significant differences between the groups (*p* < 0.05). However, data marked with the same or no letter indicate that the differences are not statistically significant (*p* > 0.05). L* represents the lightness of meat color. a* represents the redness of meat. b* represents the yellowness of meat.

**Table 6:** Effects of RPF and RPFC on the fatty acid (FA) profile and EE content in *Longissimus lumborum* of lambs.

FA, %	CON	RPF	RPFC	SEM	*p*-Value
C6:0	0.03	0.02	0.03	0.01	0.17
C8:0	0.01	0.01	0.01	0.001	0.57
C10:0	0.28	0.28	0.28	0.01	0.98
C12:0	0.30	0.35	0.38	0.04	0.36
C13:0	0.02	0.02	0.02	0.001	0.45
C14:0	5.81	6.17	6.66	0.31	0.40
C14:1	0.13	0.16	0.14	0.01	0.16
C15:0	0.62	0.58	0.65	0.03	0.38
C16:0	19.90	21.18	21.74	0.53	0.09
C16:1	2.19 ^a^	2.41 ^ab^	2.46 ^b^	0.08	0.04
C17:0	1.94 ^b^	1.62 ^a^	1.71 ^ab^	0.07	<0.01
C17:1	0.95 ^b^	0.80 ^a^	0.88 ^ab^	0.04	<0.01
C18:0	21.86	22.61	21.69	0.69	0.45
C18:1n-9t	4.78	5.25	4.28	0.28	0.24
C18:1n-9c	32.99 ^b^	30.86 ^a^	31.02 ^ab^	0.58	0.01
C18:2n-6t	0.13 ^a^	0.22 ^b^	0.12 ^a^	0.03	0.03
C18:2n-6c	4.75	4.32	4.29	0.22	0.17
C20:0/C18:3n-6	0.13	0.11	0.12	0.01	0.09
C18:3n-3	0.32	0.28	0.28	0.02	0.05
C20:2	0.04	0.04	0.04	0.003	0.30
C22:0/C20:3n-6	0.12	0.11	0.14	0.01	0.38
C20:3n-3/C22:1n-9	1.11	1.08	1.16	0.08	0.83
C23:0	0.06	0.03	0.08	0.01	0.15
C22:2/C20:5	0.03	0.03	0.03	0.003	0.12
C24:1	0.29	0.29	0.28	0.02	0.98
C22:3n-3	0.06	0.06	0.09	0.01	0.83
C22:5n-3	0.11	0.11	0.13	0.01	0.60
C22:6n-3	0.11	0.10	0.16	0.02	0.73
anteiso-C13:0	0.19	0.22	0.29	0.03	0.49
anteiso-C15:0	0.21	0.23	0.26	0.01	0.08
iso-C16:0	0.17	0.17	0.18	0.01	0.88
iso-C18:0	0.36 ^b^	0.26 ^a^	0.35 ^ab^	0.03	0.04
∑n-6 PUFA	5.14	4.75	4.68	0.23	0.25
∑n-3 PUFA	1.71	1.64	1.82	0.10	0.61
∑SFA	51.99 ^a^	53.98 ^ab^	54.61 ^b^	0.66	0.04
∑MUFA	41.33	39.87	39.07	0.57	0.06
∑PUFA	6.66	6.23	6.31	0.30	0.31
∑MUFA/∑SFA	0.79 ^b^	0.74 ^ab^	0.72 ^a^	0.02	0.04
∑PUFA/∑SFA	0.13	0.12	0.12	0.01	0.18
EE	5.42	5.49	5.41	0.11	0.63

NOTE: ∑n-6 PUFA fatty acids were calculated as the sum of C18:2n-6c, C18:2n-6t, C18:3n-6, C20:3n-6. ∑n-3 PUFA fatty acids were calculated as the sum of C18:3n-3, C20:3n-3, C22:3n-3 C22:5n-3, C22:6n-3. ∑SFA were calculated as the sum of C6:0, C8:0, C10:0, C12:0, C13:0, C14:0, C15:0, C16:0, C17:0, C18:0, C20:0, C22:0, C23:0. ∑MUFA were calculated as the sum of C14:1, C16:1, C18:1n-9c, C18:1n-9t, C22:1n-9, C24:1. ∑PUFA were calculated as the sum of C18:2n-6t, C18:2n-6c, C18:3n-3, C18:3n-6, C20:2, C20:3 n-6, C20:3n-3, C20:5, C22:3n-3, C22:5n-3, C22:6n-3. Within the same row of the table, data labeled with different lowercase letters denote statistically significant differences between the groups (*p* < 0.05). However, data marked with the same or no letter indicate that the differences are not statistically significant (*p* > 0.05).

## Data Availability

The authors affirm that all data necessary for confirming the conclusions of the article are present within the article and tables.

## References

[1] Liu H., Peng W., Mao K., Yang Y., Wu Q., Wang K., Zeng M., Han X., Han J., Zhou H. (2024). The Changes in Fecal Bacterial Communities in Goats Offered Rumen-Protected Fat. Microorganisms.

[2] Arshad U., Santos J.E.P. (2024). Graduate Student Literature Review: Exploring choline’s important roles as a nutrient for transition dairy cows. J. Dairy Sci..

[3] Enjalbert F., Combes S., Zened A., Meynadier A. (2017). Rumen microbiota and dietary fat: A mutual shaping. J. Appl. Microbiol..

[4] Huffman R.P., Stock R.A., Sindt M.H., Shain D.H. (1992). Effect of fat type and forage level on performance of finishing cattle. J. Anim. Sci..

[5] Andersen J.B., Ridder C., Larsen T. (2008). Priming the Cow for Mobilization in the Periparturient Period: Effects of Supplementing the Dry Cow with Saturated Fat or Linseed. J. Dairy Sci..

[6] Martin C., Bernard L., Michalet-Doreau B. (1996). Influence of sampling time and diet on amino acid composition of protozoal and bacterial fractions from bovine ruminal contents. J. Anim. Sci..

[7] Cartier P., Chillard Y., Paquet D. (1990). Inhibiting and Activating Effects of Skim Milks and Proteose-Peptone Fractions on Spontaneous Lipolysis and Purified Lipoprotein Lipase Activity in Bovine Milk. J. Dairy Sci..

[8] Ferguson J.D., Sklan D., Chalupa W.V., Kronfeld D.S. (1990). Effects of Hard Fats on In Vitro and In Vivo Rumen Fermentation, Milk Production, and Reproduction in Dairy Cows1. J. Dairy Sci..

[9] Hadipour A., Mohit A., Jahanian R. (2014). Effect of dietary supplementation of camel hump fat on performance, carcass characteristics, antibody responses and blood metabolites in fattening lambs. Small Rumin. Res..

[10] Pewan S.B., Otto J.R., Kinobe R.T., Adegboye O.A., Malau-Aduli A.E.O. (2022). Fortification of diets with omega-3 long-chain polyunsaturated fatty acids enhances feedlot performance, intramuscular fat content, fat melting point, and carcass characteristics of Tattykeel Australian White MARGRA lambs. Front. Vet. Sci..

[11] Nguyen D.V., Malau-Aduli B.S., Cavalieri J., Nichols P.D., Malau-Aduli A.E.O. (2018). Supplementation with plant-derived oils rich in omega-3 polyunsaturated fatty acids for lamb production. Vet. Anim. Sci..

[12] Zhou Z., Trevisi E., Luchini D.N., Loor J.J. (2017). Differences in liver functionality indexes in peripartal dairy cows fed rumen-protected methionine or choline are associated with performance, oxidative stress status, and plasma amino acid profiles. J. Dairy Sci..

[13] Bryant T.C., Rivera J.D., Galyean M.L., Duff G.C., Hallford D.M., Montgomery T.H. (1999). Effects of dietary level of ruminally protected choline on performance and carcass characteristics of finishing beef steers and on growth and serum metabolites in lambs. J. Anim. Sci..

[14] Jayaprakash G., Sathiyabarathi M., Robert M.A., Tamilmani T. (2016). Rumen-protected choline: A significance effect on dairy cattle nutrition. Vet. World.

[15] Kawas J.R., Garcia-Mazcorro J.F., Fimbres-Durazo H., Ortega-Cerrilla M.E. (2020). Effects of Rumen-Protected Choline on Growth Performance, Carcass Characteristics and Blood Lipid Metabolites of Feedlot Lambs. Animals.

[16] Li H., Hongrong W., Lihuai Y., Mengzhi W., Shimin L., Lisha S., Chen Q. (2015). Effects of supplementation of rumen-protected choline on growth performance, meat quality and gene expression in longissimus dorsi muscle of lambs. Arch. Anim. Nutr..

[17] Jin Y., Huawei L., Wang H. (2023). Dietary rumen-protected choline supplementation regulates blood biochemical profiles and urinary metabolome and improves growth performance of growing lambs. Anim. Biotechnol..

[18] Arshad U., Zenobi M.G., Staples C.R., Santos J.E.P. (2020). Meta-analysis of the effects of supplemental rumen-protected choline during the transition period on performance and health of parous dairy cows. J. Dairy Sci..

[19] Zom R.L., van Baal J., Goselink R.M., Bakker J.A., de Veth M.J., van Vuuren A.M. (2011). Effect of rumen-protected choline on performance, blood metabolites, and hepatic triacylglycerols of periparturient dairy cattle. J. Dairy Sci..

[20] Arshad U., Husnain A., Poindexter M.B., Zimpel R., Nelson C.D., Santos J.E.P. (2023). Rumen-protected choline reduces hepatic lipidosis by increasing hepatic triacylglycerol-rich lipoprotein secretion in dairy cows. J. Dairy Sci..

[21] Haddad S.G., Younis H.M. (2004). The effect of adding ruminally protected fat in fattening diets on nutrient intake, digestibility and growth performance of Awassi lambs. Anim. Feed. Sci. Technol..

[22] Jacometo C.B., Zhou Z., Luchini D., Corrêa M.N., Loor J.J. (2017). Maternal supplementation with rumen-protected methionine increases prepartal plasma methionine concentration and alters hepatic mRNA abundance of 1-carbon, methionine, and transsulfuration pathways in neonatal Holstein calves. J. Dairy Sci..

[23] Manriquez D., Chen L., Melendez P., Pinedo P. (2019). The effect of an organic rumen-protected fat supplement on performance, metabolic status, and health of dairy cows. BMC Vet. Res..

[24] Geelen S.N., Sloet van Oldruitenborgh-Oosterbaan M.M., Beynen A.C. (1999). Dietary fat supplementation and equine plasma lipid metabolism. Equine Vet. J. Suppl..

[25] Ngidi M.E., Loerch S.C., Fluharty F.L., Palmquist D.L. (1990). Effects of calcium soaps of long-chain fatty acids on feedlot performance, carcass characteristics and ruminal metabolism of steers. J. Anim. Sci..

[26] Behan A.A., Loh T.C., Fakurazi S., Kaka U., Kaka A., Samsudin A.A. (2019). Effects of Supplementation of Rumen Protected Fats on Rumen Ecology and Digestibility of Nutrients in Sheep. Animals..

[27] Marques T.C., Monteiro H.F., Melo D.B., Coelho W.M., Salman S., Marques L.R., Leão K.M., Machado V.S., Menta P., Dubey D. (2024). Effect of rumen-protected choline on dairy cow metabolism, immunity, lactation performance, and vaginal discharge microbiome. J. Dairy Sci..

[28] Chen X., Qiu W., Ma X., Ren L., Feng M., Hu S., Xue C., Chen R. (2024). Roles and Mechanisms of Choline Metabolism in Nonalcoholic Fatty Liver Disease and Cancers. Front. Biosci..

[29] Kang H.J., Piao M.Y., Park S.J., Na S.W., Kim H.J., Baik M. (2019). Effects of heat stress and rumen-protected fat supplementation on growth performance, rumen characteristics, and blood parameters in growing Korean cattle steers. Asian Australas. J. Anim. Sci..

[30] Kang H.J., Piao M.Y., Park S.J., Na S.W., Kim H.J., Baik M. (2019). Effects of ambient temperature and rumen-protected fat supplementation on growth performance, rumen fermentation and blood parameters during cold season in Korean cattle steers. Asian-Australas. J. Anim. Sci..

[31] Lytle J.R., Price T., Crouse S.F., Smith D.R., Walzem R.L., Smith S.B. (2023). Consuming High-Fat and Low-Fat Ground Beef Depresses High-Density and Low-Density Lipoprotein Cholesterol Concentrations, and Reduces Small, Dense Low-Density Lipoprotein Particle Abundance. Nutrients.

[32] de Lima J.A.M., Bezerra L.R., Feitosa T.J.O., Oliveira J.R., de Oliveira D.L.V., Mazzetto S.E., Cavalcanti M.T., Pereira Filho J.M., Oliveira R.L., de Oliveira J.P.F. (2023). Production, characterization, and dietary supplementation effect of rumen-protected fat on ruminal function and blood parameters of sheep. Trop. Anim. Health Prod..

[33] Rodríguez-Guerrero V., Lizarazo A.C., Ferraro S., Miranda L.A., Mendoza G.D., Suárez N. (2018). Effect of herbal choline and rumen-protected methionine on lamb performance and blood metabolites. S. Afr. J. Anim. Sci..

[34] Khliji S., van de Ven R., Lamb T.A., Lanza M., Hopkins D.L. (2010). Relationship between consumer ranking of lamb colour and objective measures of colour. Meat Sci..

[35] Andrade E.N., Polizel Neto A., Roça R.O., Faria M.H., Resende F.D., Siqueira G.R., Pinheiro R.S. (2014). Beef quality of young Angus×Nellore cattle supplemented with rumen-protected lipids during rearing and fatting periods. Meat Sci..

[36] Ramírez-Zamudio G.D., da Cruz W.F.G., Schoonmaker J.P., de Resende F.D., Siqueira G.R., Machado Neto O.R., Gionbelli T.R.S., Teixeira P.D., Rodrigues L.M., Gionbelli M.P. (2022). Effect of rumen-protected fat on performance, carcass characteristics and beef quality of the progeny from Nellore cows fed by different planes of nutrition during gestation. Livest. Sci..

[37] Zhang M., Zhang Z., Zhang X., Lu C., Yang W., Xie X., Xin H., Lu X., Ni M., Yang X. (2024). Effects of dietary Clostridium butyricum and rumen protected fat on meat quality, oxidative stability, and chemical composition of finishing goats. J. Anim. Sci. Biotechnol..

[38] Rexford D.R., Wallis E.S. (1947). Choline cholanate. J. Am. Chem. Soc..

[39] Siciliani D., Kortner T.M., Berge G.M., Hansen A.K., Krogdahl Å. (2023). Effects of dietary lipid level and environmental temperature on lipid metabolism in the intestine and liver, and choline requirement in Atlantic salmon (*Salmo salar* L.) parr. J. Nutr. Sci..

[40] Huo Q., Sun X., Wu T., Li Z., Jonker A., You P., Li R., Li J., Tian W., Li C. (2022). Supplementation of graded levels of rumen-protected choline to a pelleted total mixed ration did not improve the growth and slaughter performance of fattening lambs. Front. Vet. Sci..

[41] Watkins P.J., Frank D. (2019). Heptadecanoic acid as an indicator of BCFA content in sheep fat. Meat Sci..

[42] Scollan N.D., Enser M., Gulati S.K., Richardson I., Wood J.D. (2003). Effects of including a ruminally protected lipid supplement in the diet on the fatty acid composition of beef muscle. Br. J. Nutr..

[43] Razzaghi A., Valizadeh R., Naserian A.A., Mesgaran M.D., Carpenter A.J., Ghaffari M.H. (2016). Effect of dietary sugar concentration and sunflower seed supplementation on lactation performance, ruminal fermentation, milk fatty acid profile, and blood metabolites of dairy cows. J. Dairy Sci..

[44] Meng Y., Guo D., Lin L., Zhao H., Xu W., Luo S., Jiang X., Li S., He X., Zhu R. (2024). Glycolytic enzyme PFKL governs lipolysis by promoting lipid droplet-mitochondria tethering to enhance β-oxidation and tumor cell proliferation. Nat. Metab..

